# Characterization of Squeeze Film Damping in Force Rebalance Accelerometers

**DOI:** 10.3390/s26092589

**Published:** 2026-04-22

**Authors:** Hasan Baran Özmen, Melin Şahin, Gökhan Osman Özgen

**Affiliations:** 1 Roketsan A.Ş., Lalahan, Mamak, Ankara 06852, Türkiye; hasanbaranozmen@gmail.com; 2 Department of Aerospace Engineering, Middle East Technical University (METU), Cankaya, Ankara 06800, Türkiye; msahin@metu.edu.tr; 3 Department of Mechanical Engineering, Middle East Technical University (METU), Cankaya, Ankara 06800, Türkiye

**Keywords:** control system modelling, force rebalance accelerometer, open loop system modelling, closed loop system modelling, squeeze film damping, finite element modelling and analysis

## Abstract

Force rebalance-type accelerometers are mainly used in inertial navigation systems of aircraft, and the characterization of the resulting squeeze film damping (SFD) is essential for estimating dynamic response characteristics of these accelerometers. In this study, a methodology for modeling SFD and experimentally verifying this model for force rebalance-type accelerometers is presented. Modeling of the SFD effect involves determining an effective damping coefficient as a function of pendulum displacement. Damping force and pressure distribution due to SFD are obtained for a range of pendulum displacements via finite element analysis (FEA). The accelerometer is modeled as both an open- and closed-loop system, where an identified damping model for SFD is also used. The open-loop model is verified by comparing the step response of the system, and the closed-loop model is verified by comparing the frequency and shock responses of the system via simulations and experiments. Simulation and test results of both open- and closed-loop systems show close agreement. The presented results indicate that in systems with similar dimensions and material properties, damping due to SFD in a force rebalance accelerometer can be accurately modeled as a function of pendulum displacement using the method described in this research study.

## 1. Introduction

In some micro-electromechanical system (MEMS)-type inertial sensors and larger-sized force rebalance types of inertial sensors, the relative displacement of a mass element (generally called the pendulum) is sensed by measuring capacitance change using a capacitive pickoff system. This type of displacement measurement involves a stationary reference surface and the surface of the pendulum moving against it. Displacement of the pendulum results in capacitive change in the gap between the surfaces, which can be theoretically related to the relative displacement. This relationship enables measurement of displacement using measured capacitance. The relative displacement of these kinds of surfaces may also be used to generate what is called capacitive forcing in certain types of sensor designs. In order to increase the sensitivity of the capacitive pickoff or the capacitive forcing (i.e., capacitance change per unit displacement or force), the nominal air-gap distance between the moving pendulum and reference stationary surfaces needs to be as small as possible. When the relative motion between the surfaces is dynamic, air (or whatever gas is used in the sensor design) present in the gap region is forced to flow out, or air/gas from outside is sucked into the gap region. This type of air/gas motion causes a rise or a drop in air/gas pressure in the gap. In either case, a resistive type of equivalent force is formed on surfaces moving relative to each other, opposing the relative motion. This force (which acts like a damping force) is caused by the so-called squeeze film damping (SFD) effect and becomes a significant factor for pendulum motion for cases when the ratio of the width of the moving plate to the air gap is bigger than three [1]. In this paper, a methodology for experimental identification and verification of the SFD present in a force rebalance-type accelerometer is developed. Displacement dependency of the SFD is included in both the modeling and identification work presented. The force rebalance-type accelerometers studied in this paper are typically used in inertial navigation systems of aircraft and marine vessels.

SFD caused by the air/gas in between the moving pendulum and reference stationary surfaces mostly originates from inertial and viscous effects. Inertial effects are generally neglected, and viscous effects remain the main source of damping in both the MEMS-type inertial sensors and force rebalance-type inertial sensors due to their small-scale geometry. The effects of SFD on the operational performances of MEMS inertial sensors and larger-sized force rebalance-type inertial sensors vary. For these sensors, input–output frequency response and shock response characteristics are two of the main metrics that determine dynamic behaviors of such systems. If the mechanical structure of the sensor is designed correctly, providing proper SFD in the system, this may help to achieve the desired frequency response characteristics of the sensor. Also, the right amount of damping coming from the SFD effect assures protection against high-amplitude mechanical shocks. On the other hand, most of the MEMS devices operate on the principle of vibration at the resonant frequency of the moving structure. In such oscillatory systems, a high-quality factor is desired, which is inversely related to damping in the system. Therefore, it is essential to reduce SFD as much as possible in these types of MEMS sensors.

Great effort is devoted to the modeling and characterization of SFD in various devices such as accelerometers, optical switches, and micro actuators. A comprehensive and general overview of the SFD for MEMS devices, including modeling and analysis methods, is presented in [1] and [2]. In some of the MEMS devices, transversely moving perforated plates are preferred in order to reduce SFD [3,4,5,6,7]. In some micro- and nanoscale resonator structures working in a low ambient pressure environment, gas rarefaction effects are not negligible and have to be considered in SFD modeling [1,8,9,10,11,12]. The concept of the squeeze number is introduced in [13], providing information about gas compressibility. Gas behaves as a damper in systems with low squeeze numbers, whereas at high squeeze-number values, it cannot escape between two surfaces, and, therefore, it squeezes and starts to act like a spring [14]. In cases of displacement of moving surfaces that are comparable to the nominal air gap, such amplitude effects at low squeeze numbers are analytically modeled [13,14,15]. There are some studies that focus on calculating the moving plate’s effective lateral dimensions to use them in analytical models instead of the original dimensions [16,17,18,19,20]. A complete model comprising both border and amplitude effects is proposed in [21]. Moeenfard [22] investigates SFD in torsional micro actuators. Flexible microstructures under SFD are analyzed by Nayfeh and Younis [23]. Simple moving plate shapes are commonly modeled in the literature. On the other hand, nontrivial geometries are modeled by using FEM. In [24], both damping and noise terms due to SFD are investigated. Chen and Kuo [25] propose a numerical procedure to model squeeze and viscous damping in micro electrostatic comb drives. Sun et al. [26] investigate the effects of SFD on the switching time of a micro-scaled optical switch. Mo et al. [27] proposed an open-loop test method to characterize damping due to squeeze film effects in MEMS accelerometers. In [28], SFD in nonlinear capacitive circular microplates is modeled and characterized experimentally. Another study on the analysis of SFD in MEMS structures is by Nayfeh and Younis [29], in which squeeze film damping forces are modeled for parallel microplates where flexural deformations of one of the plates are also taken into account. Modeling is done analytically, and the damping characteristics estimation of the flexible plate is performed and studied. The results are compared to experimental findings available from the literature. The study is not specifically intended for an accelerometer, but rather more for actuator applications, and the surfaces subject to SFD are parallel and the relative motion between them is not rotational. Fang et al. [30] examined the relationship between cross-axis sensitivity (sensitivity to axes perpendicular to the axis of reading) and SFD for a proof mass connected to four built-in beams of MEMS size. It was determined that the SFD-induced torque had a reducing effect on cross-axis sensitivity, and analytical modeling and FEA results were compared. Lu et al. [21] worked on analytical modeling of SFD considering border effect and amplitude effect for sensitive MEMS-type devices. Experimental and analytical results were also compared. Taghavi et al. [31] studied the modeling and analysis-based design of a closed-loop MOEMS accelerometer with no effort on modeling SFD.

Application-oriented works studying SFD in the literature reviewed in this paper are all related to MEMS-type devices, with some on MEMS-type capacitive sensors and some on MEMS-type actuators. The structural design of the components subject to SFD is of a parallel flat plate type [8,9,10,21,22,26,27,29], while for some of them, these components are of a perforated plate type [3,4,5,6,11]. Most of these studies consider only small amplitudes (i.e., plate displacements being very small compared to the air gap between surfaces) with SFD effects modeled analytically [4,5,6,8,9,10,11,17,22,25,26,27,29], using the finite element method [3,4,6,7,8,10,11,27], and experimentally [3,7,27]. In some of the studies, rarefaction effects [8,9,10,11,17] and surface roughness effects [9] are also taken into account. Very few of the reviewed works consider large displacements [21,23]. Among these, Qianbo et al. [21] study SFD for parallel flat plates considering large displacements both analytically and experimentally. Younis and Nayfeh [23] study SFD forces analytically for parallel microplates where flexural deformations of one of the plates and large deflections and actuation loads are also taken into account. Pendulum-type structural design is only studied by Mo et al. [27] using the finite element method, along with experimental verification of results for the open-loop use of the MEMS-type sensor being studied, but only considering small displacements. Considering the reviewed works, it can be stated that this paper is unique in its handling of the issue of SFD, which involves the pendulum-type asymmetrical partial annular plate configuration in force balance-type accelerometers considering large displacements. Also, the paper validates the finite element method-based modeling and analysis of SFD in the aforementioned conditions using both open-loop and closed-loop fully scaled operational system models of the accelerometer being studied. The experimentally verified high-fidelity model used for modeling the force balance-type accelerometer considering SFD is also unique considering the published works on the general subject matter.

Force balance types of accelerometers are often used to measure acceleration in static and quasi-static (i.e., to detect rigid body motions) as well as dynamic situations, which necessitates accurate measurement of acceleration in a broad frequency range as well as displacement range. Damping present in the accelerometer as a structural system and acting on the pendulum element affects accelerometer performance and accuracy in both aspects. Typically, force rebalance accelerometers are expected to provide an effective bandwidth of 400 Hz to 500 Hz, per the requirements of the motion control systems (i.e., guidance systems) in which they are frequently used. The effective bandwidth of the accelerometer is generally determined by the value of frequency at which the frequency response function (FRF) of the accelerometer (ratio of the harmonic amplitudes of the input acceleration and the measured acceleration) has a 3 decibel deviation from unity in magnitude or 90 degree deviation in phase angle as it approaches its resonant frequency. The platforms on which they are mounted also commonly experience vibrational responses up to 2000 Hz; thus, it is also desirable to characterize the frequency-dependent dynamic response characteristics of the accelerometer up to this frequency. The damping forces acting on the pendulum of the accelerometer significantly affect the FRF defining the input acceleration and measure the acceleration relationship of the sensor as the frequency is approaching the resonant frequency of the accelerometer. This makes accurate modeling of SFD, which is the major source of damping for force balance-type accelerometers, a must in order to estimate the frequency-based response of the sensor acceleration measurement when designing these types of accelerometers. Another issue about the damping introduced by SFD is that it affects the amount of displacement of the pendulum around the resonant frequencies of the accelerometer when it is subject to broadband acceleration inputs. Making the pendulum displace less ensures that the system operates in the linear region, which makes SFD-based damping somewhat desirable. Failure to model SFD-based damping correctly in the design phase may lead to an accelerometer design that may not be able to provide the required bandwidth. Also, outside of the bandwidth of the accelerometer, the high-frequency inputs also potentially cause large displacements around resonant frequencies that may cause the pendulum to come into contact with the metal capacitive surfaces it is facing, leading to sensor saturation or mechanical damage. The damping effect provided by SFD also controls these resonant responses of the pendulum element outside of its effective bandwidth. In order to tune the damping provided by the SFD such that the desired bandwidth is achieved, with the resonant vibration response of the pendulum in check, accurate modeling of the SFD is required. While analytical models exist for modeling SFD in MEMS-type systems where capacitive surfaces move linearly in the normal direction relative to each other, in force rebalance accelerometers, capacitive surfaces rotate relative to each other, causing the gap to become variable along the surfaces, making it hard to come up with an analytical closed-form solution for SFD-based damping forces. This issue, combined with the partial annular shape of the pendulum capacitive surfaces, makes finite element method-based modeling of the SFD effect a practical alternative. Once a modeling approach is validated for modeling SFD in force rebalance accelerometers, this model can be used for tuning the damping in the system for the desired dynamic performance of the accelerometer by using the shape and dimensions of the capacitive surfaces and the distance between the pendulum and the capacitive surfaces as design parameters.

Despite the extensive literature on squeeze film damping (SFD) in MEMS devices, most existing studies are limited to parallel plate configurations, small displacement assumptions, and translational motion. In contrast, the present work focuses on a force rebalance accelerometer with dimensions larger than typical MEMS devices, featuring a pendulum-type partial annular geometry in which the relative motion is rotational and results in a non-uniform gap distribution. Furthermore, large displacement effects are incorporated into the SFD modeling. Within this scope, SFD acting on the moving pendulum structure is modeled using a finite element method (FEM)-based approach, and the dynamic response of the pendulum is represented as a single-degree-of-freedom system with displacement-dependent damping. The developed SFD model is integrated into full-scale accelerometer models in both open-loop and closed-loop configurations. Experimental setups are designed to obtain the step response in open-loop operation and the input–output frequency response and mechanical shock response in closed-loop operation. The proposed modeling approach is then validated by comparing the simulation and experimental results. To the best of the authors’ knowledge, such a configuration—combining a partial annular pendulum geometry, rotational motion, and large displacement effects—has not been comprehensively modeled and experimentally validated in the existing literature. Therefore, the present study represents a novel contribution specifically for force rebalance accelerometers employing this class of geometry.

## 2. Description of Components and Working Principle of the Force Rebalance Accelerometer

**Figure 1:**
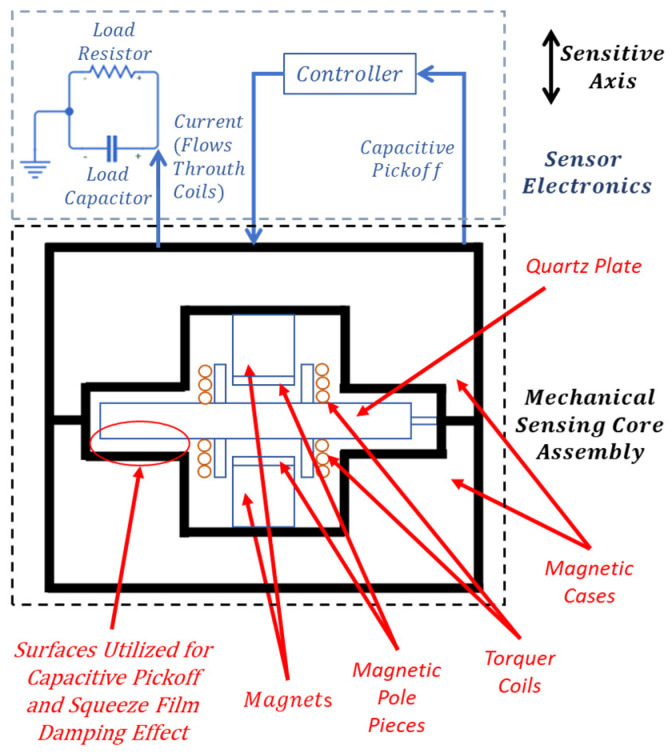
Schematic of the accelerometer system.

**Figure 2:**
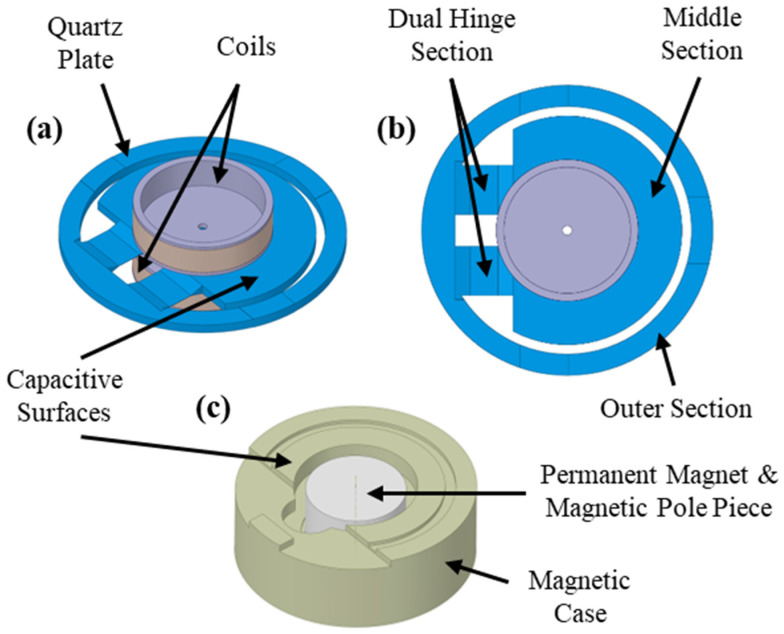
(**a**) Isometric view of quartz plate–coils assembly: (**b**) top view of quartz plate–coils assembly and sections of quartz plate, (**c**) isometric view of magnetic case assembly.

The force rebalance accelerometer analyzed in this study is a single-axis, non-gyroscopic inertial sensor designed for translational acceleration sensing, and the detailed working principle of this type of accelerometer is given in [32,33]. In this section, the components and main working principle of the specific type of accelerometer investigated in this study are briefly defined. As illustrated in Figure 1, the system consists of a mechanical sensing core and integrated electronics. The core assembly features a quartz plate rigidly connected to torquer coils on both sides (Figure 2a,b), positioned between two magnetic case assemblies—each comprising a case, magnet, and pole piece (Figure 1 and Figure 2c). Linked to this core, the sensor electronics incorporate the capacitive pickoff demodulator and controller components (Figure 1). The material of the pendulum, quartz, and the material of the magnetic cases, special nickel–iron alloys, have low coefficients of thermal expansion properties [33].

The quartz plate–coils assembly consists of a wet-etched quartz plate with two attached coils (Figure 2a,b) whose concentric alignment is critical for ensuring symmetric force generation, minimizing bias errors, and reducing cross-axis sensitivity. This monolithic quartz component features a middle section which forms a pendulum with two coils attached to it (together with bonding material), an outer section for magnetic case connection, and a dual hinge section that joins them (Figure 2b). While the middle and outer sections share a rigid thickness of 600–800 µm, the hinges are significantly thinner (15–25 µm). Consequently, these flexible hinges allow the pendulum to undergo angular displacement relative to the magnetic cases when subjected to an acceleration normal to the plate surface.

The conductive-coated surfaces of the quartz plate’s middle section and the opposing magnetic cases form two capacitor elements (Figure 2a,c). These capacitors, integrated with the sensor electronics’ demodulator, constitute a capacitive sensing arrangement that monitors pendulum displacement. Under zero acceleration, the capacitance values remain balanced due to the lack of deflection. However, acceleration-induced displacement creates a capacitive difference, which the demodulator detects and converts into a signal for the controller.

As shown in Figure 2c, the magnetic case assembly consists of a permanent magnet flux source, a soft magnetic alloy case, and a pole piece to direct the flux. This assembly generates a specific magnetic flux density across the torquer coils. When a capacitive difference is detected, the controller supplies a current to these coils, generating a Lorentz force that restores the pendulum to its nominal position. Simultaneously, this feedback current is exported as an external signal and measured as a voltage across a load resistor, often filtered by a load capacitor. Consequently, the accelerometer produces an electrical output directly proportional to the experienced acceleration.

**Figure 3:**
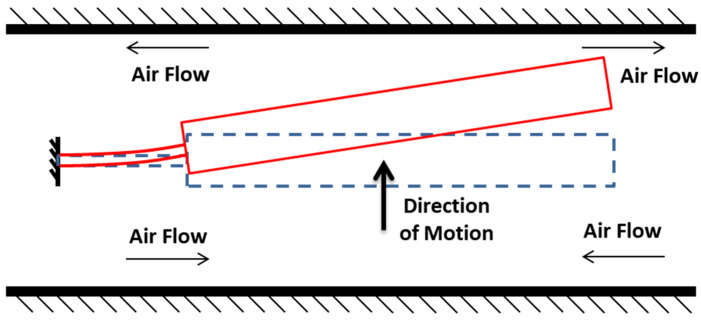
Fluid flow during motion.

System resolution improves as the gap between the pendulum and magnetic case capacitive surfaces decreases; thus, designs typically utilize 15–25 mm pendulum dimensions with narrow 15–25 µm air gaps. As the pendulum moves, air is displaced from this gap, causing pressure fluctuations that generate squeeze film damping (SFD) (Figure 3). Because the surface area significantly exceeds the gap width, SFD becomes the dominant damping force [1]. Specifically, as the gap narrows, viscous forces increase, providing essential damping to the system [1]. This fluid behavior and the resulting pressure distribution are governed by the Reynolds equation for thin films [1].

## 3. Modeling of the Force Rebalance Accelerometer with SFD

In this section, models developed for the components forming the force rebalance accelerometer, as well as the SFD, are presented. These components are the quartz plate–coils assembly, the magnetic feedback torquer, sensor electronics, and the readout circuit. SFD modeling is presented as a separate subsection where the damping force on the pendulum is modeled considering squeeze film phenomena. The models presented for these components, and SFD, will later be combined to construct full-scale sensor models for open-loop and closed-loop operational modes of the accelerometer.

### 3.1. Modeling of Quartz Plate–Coils Assembly

**Figure 4:**
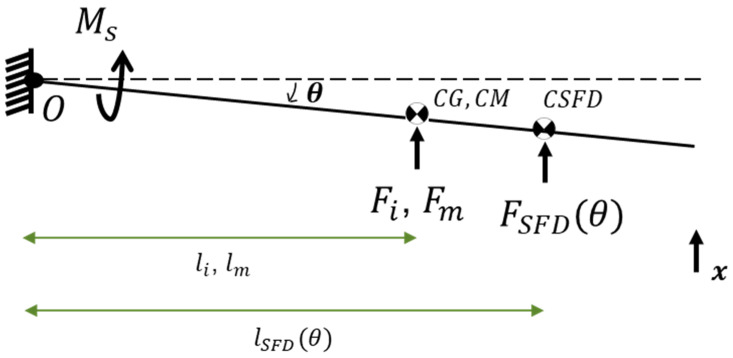
Forces acting on pendulum structure (note that coils are not shown).

In this study, the pendulum structure is assumed and modeled as a rotational, single-degree-of-freedom system. Forces acting on the pendulum structure are shown in Figure 4, where θ is the rotation of the pendulum about point O, $M_{s}$ is the recovery spring torque coming from an equivalent torsional spring at point O, $F_{SFD}(\theta )$ is the force due to the SFD, $F_{i}$ is the inertial force, $F_{m}$ is the magnetic force, CG is the center of gravity of the pendulum, CM is the center of the magnetic feedback force, CSFD is the center of pressure due to the squeeze film effects, $l_{i}$ is the moment arm length of $F_{i}$, $l_{m}$ is the moment arm length of $F_{m}$, and $l_{SFD}(\theta )$ is the moment arm length of $F_{SFD}\left(\theta \right).$ In an ideal design, CG and CM are identical. Pressure distribution on the pendulum caused by SFD changes with pendulum displacement, and thereby $F_{SFD}(\theta )$ and $l_{SFD}(\theta )$ change and torque due to SFD also changes. Because of that, $F_{SFD}(\theta )$ and $l_{SFD}(\theta )$ are modeled as functions of rotational displacement, θ.

The equation of motion of the pendulum structure and rotational stiffness coefficient can be written as

(1) $$I_{O}\ddot{\theta }+C_{r}(\theta )\dot{\theta }+K_{r}\theta =F_{m}(I)l_{m}+F_{i}l_{i}$$

(2) $$K_{r}=\frac{2E\left(\frac{1}{12}w_{hinge}h_{hinge}^{3}\right)l_{CG}}{\left({\left(l_{hinge}+l_{CG}\right)}^{2}-l_{CG}^{2}\right)}$$

In Equation (1) $I_{O}$, $C_{r}(\theta )$ and $K_{r}$ are the moment of inertia about point O, rotational damping, and rotational stiffness coefficients, respectively. In Equation (2), E, $w_{hinge}$, $h_{hinge}$, $l_{CG}$ and $l_{hinge}$ are the Young’s modulus of the quartz plate, total width and height of the dual hinge structure, distance from the center of gravity of the pendulum to the tip of the hinges, and length of the dual hinge structure, respectively. $K_{r}$ is obtained using an analytical model, which is based on beam deflection analysis, where the dual beam hinge structure is modeled as a slender elastic beam. Moment of inertia $I_{o}$ of the pendulum can be determined using CAD software.

(3) $$M_{SFD}=F_{SFD}\left(\theta \right){\dot{x}}_{SFD}(\theta )=c_{SFD}(\theta ){\dot{x}}_{SFD}^{2}(\theta )=c_{SFD}(\theta )l_{SFD}^{2}(\theta )\dot{\theta }$$

where ${\dot{x}}_{SFD}(\theta )$ is the translational velocity of the center of $F_{SFD}\left(\theta \right)$. From Equation (3) and Equation (1), the rotational damping coefficient due to the squeeze film effect can now be defined as

(4) $$C_{r}(\theta )=c_{SFD}(\theta )l_{SFD}^{2}(\theta )$$

Inertial force in Equation (1) can be defined as

(5) $$F_{i}=ma$$

where m is the mass of the pendulum and a is the acceleration that the accelerometer is experiencing along its main axis of measurement (shown as the x-axis in Figure 4). Regarding the issue of CG and CM being tuned to be in the same position, it should be noted that the force rebalance type accelerometer aims to measure inertial acceleration using the response of the pendulum dominated by its first mode shape. While the first mode of the pendulum is around 10–30 Hz, there are second and third modes below 400 Hz. The misalignment of CG and CM will cause a couple moments to occur on the pendulum, which will generate responses from the second and third modes (second bending and first torsional modes) of the accelerometer, reducing the dynamic performance (i.e., accuracy) of the accelerometer. In addition, with the triggering of the second and third modal responses, deformations will occur at the free edges of the pendulum even in equilibrium, and there will be a risk of these edge lines touching the metal capacitive surfaces opposing the pendulum surfaces.

### 3.2. Modeling of Squeeze Film Damping (SFD)

Capacitive surfaces on the pendulum and the magnetic case are shown in Figure 2a. SFD also takes place on these surfaces as the pendulum moves with a certain velocity. Since the displacement of the pendulum is very small, the intersection of the pendulum and magnetic case capacitive surfaces is considered as the effective area for the distributed damping force generated by SFD, which is given in Figure 2b.

In this study, the translational damping coefficient and geometrical location of the pressure distribution due to squeeze film effects will be obtained as a function of angular displacement of the pendulum using FEM to include amplitude effects in the modeling of SFD. Before describing the details of finite element modeling and analysis procedure, issues like rarefaction, compressibility, pressure boundary condition, and amplitude effects are discussed.

Rarefaction effects, involving a reduction in gas viscosity at low ambient pressures [13,14,15,16], are governed by the Knudsen number, $K_{n}$:

(6) $$K_{n}=\lambda /h_{0}$$

where λ is the mean free path of molecules and $h_{0}$ is the nominal air film thickness. Since the ambient mean free path ($\lambda_{0}=0.064\;\mu m$) is significantly smaller than the air gap of the system studied under atmospheric conditions, $K_{n}$ remains below 0.01. Consequently, rarefaction is considered negligible, justifying the use of standard dynamic viscosity [2]. Similarly, gas compressibility is evaluated via the squeeze number (σ) [1]:

(7) $$\sigma =\frac{12\mu \omega l^{2}}{P_{a}h_{0}^{2}}$$

where *μ* is the viscosity of the gas, *ω* is the frequency of the motion in radians per second, *l* is the typical length of the plate, and $P_{a}$ is the ambient pressure. In this study, σ is estimated to be less than 0.2, considering the maximum operational frequency of 2000 Hz and the anticipated pendulum displacement. Thus, compressibility and associated elastic forces are neglected, with SFD (Figure 3) modeled strictly as a damping mechanism [2].

Regarding boundary conditions, the finite element (FE) model prescribes zero relative pressure at the plate periphery, an assumption validated by high lateral-dimension-to-gap ratios [1]. Large-amplitude oscillations conventionally require a correction factor:

(8) $$f_{d}(\epsilon )=\frac{1}{{\left(1-\epsilon^{2}\right)}^{3/2}}$$

where the displacement ratio is $\epsilon =h/h_{0}$; h and $h_{0}$ are the displacement of the moving surface and the nominal air gap between the moving and stationary surfaces. This study employs FE-based analysis over discrete angular positions to inherently capture displacement dependency, thereby precluding the need for Equation (8).

**Figure 5:**
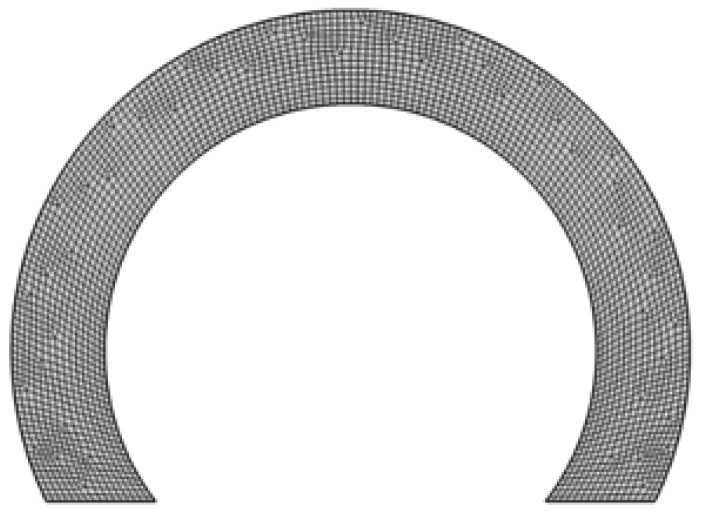
Mesh structure of the partial annular plate.

The finite element modeling of SFD for the pendulum structure is done using the thin-film flow module of the software package COMSOL Multiphysics^®^ v.6.0 [34]. This software is used to model and analyze SFD on the pendulum surface. The moving surface is modeled in 2D, and the meshed geometry is given in Figure 5. Free Quad-type elements with about 0.25 mm edge size are used. The mesh size is selected to include a large number of elements across the smallest dimension.

**Figure 6:**
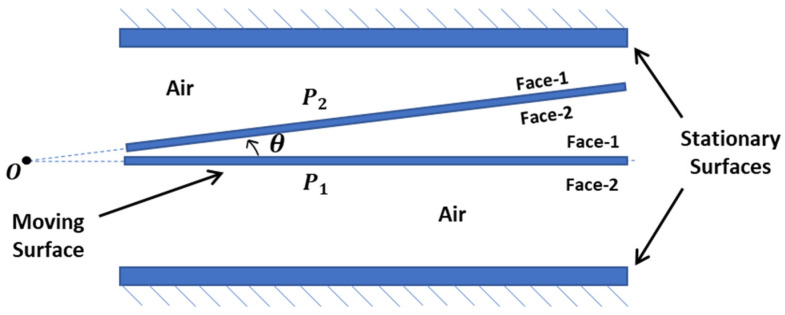
Schematic representation of the cross-sectional view of the mechanical sensing core assembly.

Figure 6 illustrates the cross-sectional schematic of the analysis model. The pendulum’s moving surfaces, designated as Face-1 and Face-2, are positioned between two stationary capacitive surfaces representing the magnetic cases. To derive the rotational damping coefficient as a function of displacement, the motion of these surfaces is defined parametrically relative to the angular displacement. The gas medium is modeled as air at ambient temperature and atmospheric pressure. The interaction between the surfaces and the gas is governed by the Modified Reynolds Equation, with boundary conditions set to zero relative pressure at the plate edges. The moving surfaces are assumed to undergo rigid body sinusoidal motion along the surface normal. A parametric step-sine analysis was conducted in the frequency domain under small-amplitude assumptions. Pressure distributions were evaluated for angular displacements ranging from 0 to 1.2 mrad across frequencies of 100 Hz, 500 Hz, 1000 Hz, and 2000 Hz to characterize frequency-dependent SFD effects.

**Figure 7:**
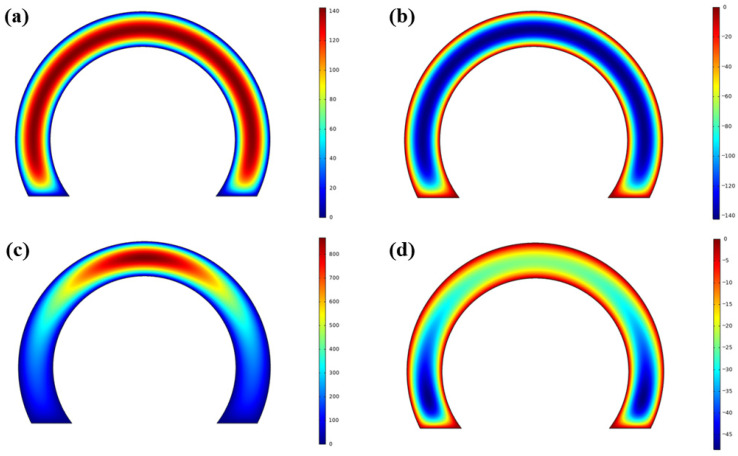
Pressure distribution on the pendulum plate: (**a**) Face-1 for position $P_{1}$, (**b**) Face-2 for position $P_{1}$, (**c**) Face-1 for position $P_{2}$, (**d**) Face-2 for position $P_{2}$.

In Figure 7a–d, pressure distributions on both faces for two different values of θ are given (position labeled as *P*_1_, where θ is zero and position labeled as *P*_2_, where θ is larger than 0) for the 100 Hz case. Note that the color scales in this particular figure are independently normalized in each subplot for better illustration of the spatial variations. Consequently, the ranges differ between subfigures, and quantitative comparisons should be made with reference to the individual color bars. It is observed that the motion of the moving plate causes SFD on both sides of it (Face-1 and Face-2). When the plate moves towards the top stationary surface, positive pressure develops on Face-1, and negative pressure develops on Face-2. The total force on the moving plate is equal to the resultant of the distributed forces (i.e., pressure distribution) acting on these two surfaces.

As can be seen in Figure 7a,d, the pressure distribution on the surfaces changes as the angular deflection θ changes. This also means that the geometrical center of the distributed pressure changes as the angular deflection θ changes. For the position $P_{1}$ (i.e., θ being equal to zero), the pressure is zero along the border, and it increases towards the center of the surface. Also, pressure magnitudes are the same for Face-1 and Face-2. On the other hand, for the position $P_{2}$ (i.e., θ is larger than zero), the distance between moving and stationary surfaces is not constant along the surface; therefore, the pressure on Face-1 increases towards the tip of the pendulum, and it becomes dominant in comparison to the pressure on Face-2.

**Figure 8:**
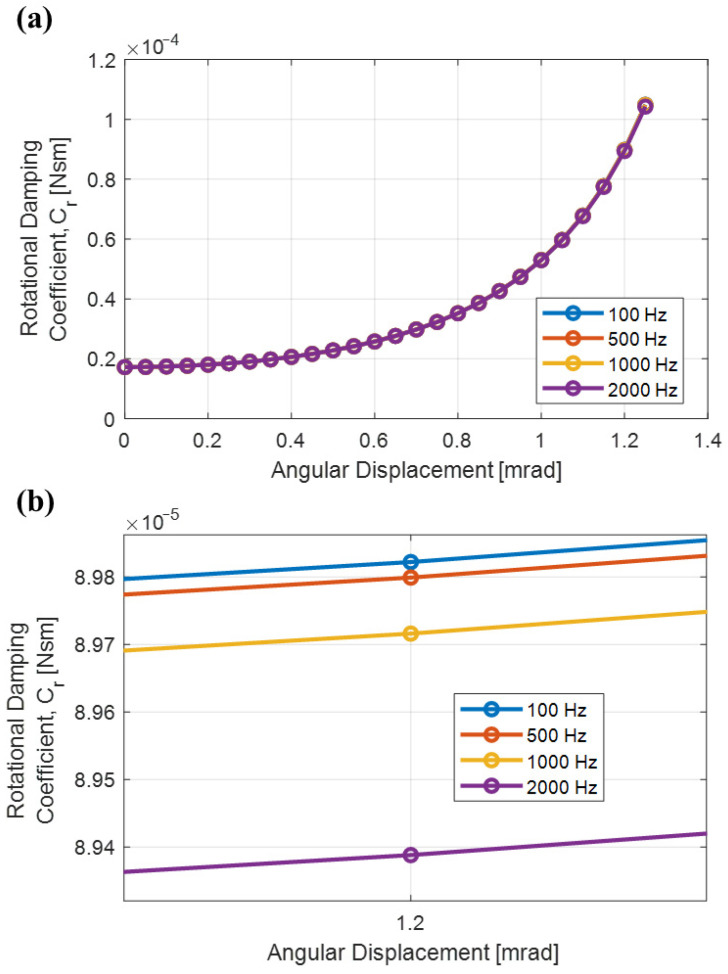
Rotational damping coefficient vs pendulum angular displacement: (**a**) rotational damping coefficient vs pendulum angular displacement plots for pendulum motion input applied at different frequencies, (**b**) rotational damping coefficients for 1.2 mrad peak pendulum angular displacement amplitude for pendulum motion input applied at different frequencies.

Using the results of pressure distribution, the translational damping coefficient can be obtained as a function of angular deflection. The geometrical center of pressure due to SFD for each value of θ is calculated, and the location of the effective damping force for each value of θ is obtained. Then, the rotational damping coefficient $C_{r}(\theta )$, which is presented in Figure 8a, is calculated for θ ranging from 0 mrad to 1.2 mrad using Equation (4). Note that $C_{r}(\theta )$ changes less than 0.1% for frequencies below 100 Hz and 1% between frequencies 100 Hz through 2000 Hz, as can be demonstrated explicitly in Figure 8b; therefore, $C_{r}(\theta )$ can be modeled independently from frequency. A polynomial function is fitted to the discretely calculated rotational damping coefficient $C_{r}(\theta )$ data obtained at 100 Hz, which will later be used in the system-level model of the accelerometer design studied in this paper.

### 3.3. Modeling of Magnetic Feedback Torquer Coils

Magnetic feedback torquer coils (Figure 1 and Figure 2) provide a closed-loop rebalance force to counteract inertial forces acting on the pendulum during acceleration. These coils are integrated into the quartz plate assembly and situated within the magnetic case air gaps. In each assembly, magnetic flux originates from a permanent magnet and returns through the magnetic case, ensuring a constant magnetic flux density across the coils. When current passes through the torquer coils, it generates a Lorentz force, expressed as

(9) $$F_{m}\left(I\right)=2BIl_{c}$$

where B, $l_{c}$ and I denote the magnetic flux density on each coil, the length of each coil, and the current flowing through each coil, respectively. The term $F_{m}\left(I\right)$ denotes the combined magnetic force of both coils. For modeling purposes, B is determined experimentally. In an ideal design, the center of gravity (CG) and the center of magnetic forces (CM) are coincident, resulting in equal moment arms ($l_{i}=l_{m}$). Consequently, the inertial and magnetic forces are assumed to be equal at steady state. By equating Equations (5) and (9), B can be derived as follows:

(10) $$B=\frac{ma_{i}}{2Il_{c}}$$

The acceleration output of the sensor is obtained as a potential difference $v_{L}$, by using a load resistor $R_{L}$. Therefore, Equation (10) is modified as

(11) $$B=\frac{ma_{i}}{2\left(\frac{v_{L}}{R_{L}}\right)l_{c}}$$

where $v_{L}$ is obtained by the accelerometer when the input acceleration $a_{i}$ is equal to gravitational acceleration. Since the values of m, $R_{L}$ and $l_{c}$ are known by design, B is obtained by using Equation (11).

### 3.4. Modeling of Sensor Electronics

Sensor electronics consist of a capacitive pickoff demodulator and controller circuits. The pickoff detects pendulum displacement, which generates capacitive asymmetry within the system. The capacitance of these parallel plates is defined by Equation (12).

(12) $$C=\epsilon \frac{A}{d}$$

where ϵ, A and d denote the dielectric permittivity, surface area, and gap distance, respectively. The relationship between the pendulum displacement and capacitance is inherently nonlinear due to the inverse dependence on d. This nonlinearity is further compounded by the pendulum’s rotational motion rather than its translational motion. The resulting relationship between the pickoff output signal and angular displacement is expressed as

(13) $$v_{po}=\theta K_{po}(\theta )$$

where $v_{po}$ and $K_{po}(\theta )$ represent the capacitive pickoff output signal and its displacement-dependent gain, respectively. This gain, modeled to account for capacitive nonlinearities, is derived from open-loop experimental measurements detailed in Section 3.5. The signal $v_{po}$ is processed by a PID controller, whose output $v_{c}$ generates the feedback current for the torquer coils to produce the rebalance force. The time domain relationship between the pickoff signal and the controller output is defined as

(14) $$v_{c}\left(t\right)=K_{P}v_{po}\left(t\right)+K_{i}\int^{t}v_{po}\left(t\right)dt+K_{d}\frac{dv_{po}\left(t\right)}{dt}$$

where $K_{p}$, $K_{i}$ and $K_{d}$ are proportional, integral, and derivative constants of the controller.

### 3.5. Modeling of Torquer Coils and Readout Circuit

**Figure 9:**
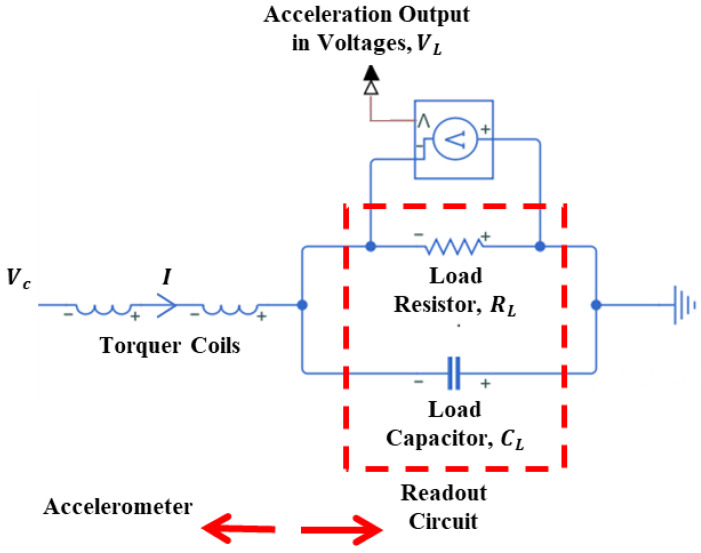
Schematic representation of torquer coils and readout circuit.

The readout electronics, integrated with the torquer coils as shown in Figure 9, convert the accelerometer’s output current into a voltage signal ($V_{L}$) through a load resistor ($R_{L}$). In this closed-loop architecture, the current-per-unit acceleration remains invariant to changes in $R_{L}$. However, while increasing $R_{L}$ enhances voltage sensitivity, it simultaneously constrains the measurement range due to electronic saturation. This saturation limits the feedback current available to counteract high acceleration loads; thus, reducing $R_{L}$ is necessary to extend the operational range. Furthermore, $R_{L}$ serves as a critical design parameter for the system’s dynamic response. When coupled with a load capacitor ($C_{L}$), it functions as a low-pass filter for the sensor output (Figure 9). The Laplace domain relationships governing $V_{C}$, I, and $V_{L}$ are defined in Equations (15) and (16):

(15) $$I(s)=\left(\frac{sR_{L}C_{L}}{s^{2}R_{L}C_{L}L_{t}+s\left(R_{L}C_{L}R_{t}\right)+(R_{t}+R_{L})}\right)V_{c}(s)$$

(16) $$V_{L}\left(s\right)=\left(\frac{R_{L}}{sR_{L}C_{L}+1}\right)I(s)$$

where $R_{t}$ and $L_{t}$ are the total resistance and total inductance of two coils, respectively.

## 4. Modeling of the Accelerometer as an Open-Loop System

In the normal operation mode of the force rebalance accelerometer, the system is operated as a closed-loop system, where a controller is utilized to automatically keep the pendulum position at its nominal point. If the controller in the accelerometer is not used and the torquer coils are driven manually, allowing the alteration of the angular position of the pendulum, this may be described as using the force rebalance accelerometer as an open-loop system. Step responses of this open-loop system to different levels of external current excitations can be utilized to validate the displacement-dependent damping coefficient (Figure 8) obtained using the FEA conducted in Section 3. Also, capacitive pickoff gain, $K_{po}(\theta )$, can be characterized using the steady state response of the open-loop test prototype to the different magnetic force inputs.

**Figure 10:**
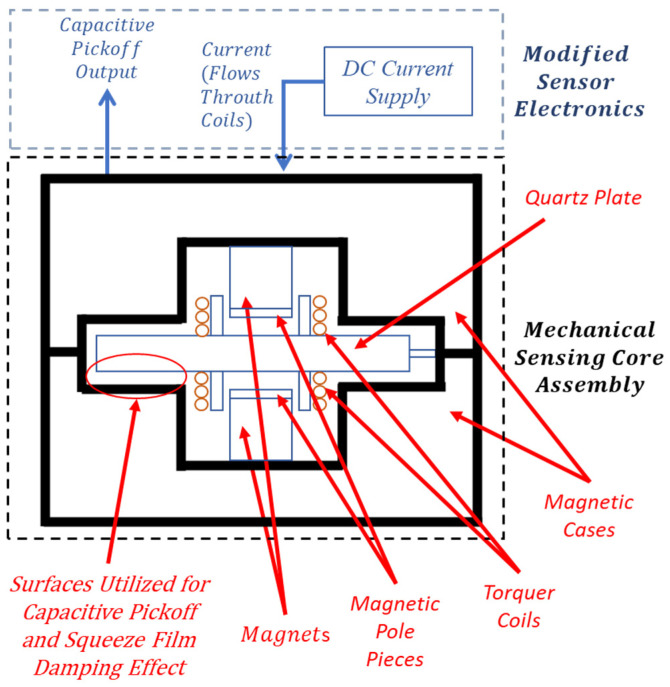
Schematic of the accelerometer operated as an open-loop system.

In order to use the force rebalance accelerometer in open-loop mode for the aforementioned experiments, the sensor electronics of a prototype accelerometer will be modified, and the mechanical sensing core assembly will be used with sensor electronics without the controller. The modified accelerometer to be used in open-loop mode is given schematically in Figure 10. In order to operate the accelerometer in an open-loop manner, the controller is not used in the model. The magnetic feedback torquer structure is excited externally by a predefined current input to the torquer coils. The output of the capacitive pickoff signal from the demodulator is obtained as a voltage signal and converted to angular displacement.

**Figure 11:**
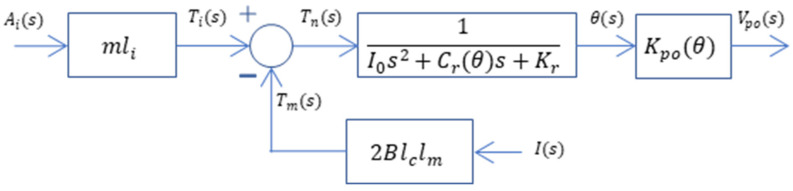
Block diagram of the accelerometer operated as an open-loop system.

Block diagram representation of the accelerometer operated as an open-loop system is given in Figure 11, where $A_{i}\left(s\right)$ is the acceleration the sensor experiences, I(s) is the current externally supplied to the torque coils, $T_{i}\left(s\right)$ is the torque due to inertial resistance, $T_{m}\left(s\right)$ is the torque due to the magnetic force, $T_{n}\left(s\right)$ is the net torque on the pendulum, Θ(s) is the angular displacement of the pendulum, and $V_{po}\left(s\right)$ is the output of the capacitive pickoff, with all defined in the Laplace or s domain. The term $K_{po}(\theta )$ in the block diagram is the gain of the capacitive pickoff and defines the relationship between the angular displacement and the electronic output of the pickoff demodulator in the sensor electronics. This parameter is not a constant gain term, and it depends on the instantaneous value of the angular displacement, since the relation between $V_{po}\left(s\right)$ and Θ(s) is not linear. The term $C_{r}(\theta )$ in the model also depends on the instantaneous value of angular displacement. The software package MATLAB^®^ 2022b and its Simulink^®^ toolbox will be used to run simulations, which are presented next.

### Experimental Verification of the Open-Loop System Model of the Accelerometer

**Figure 12:**
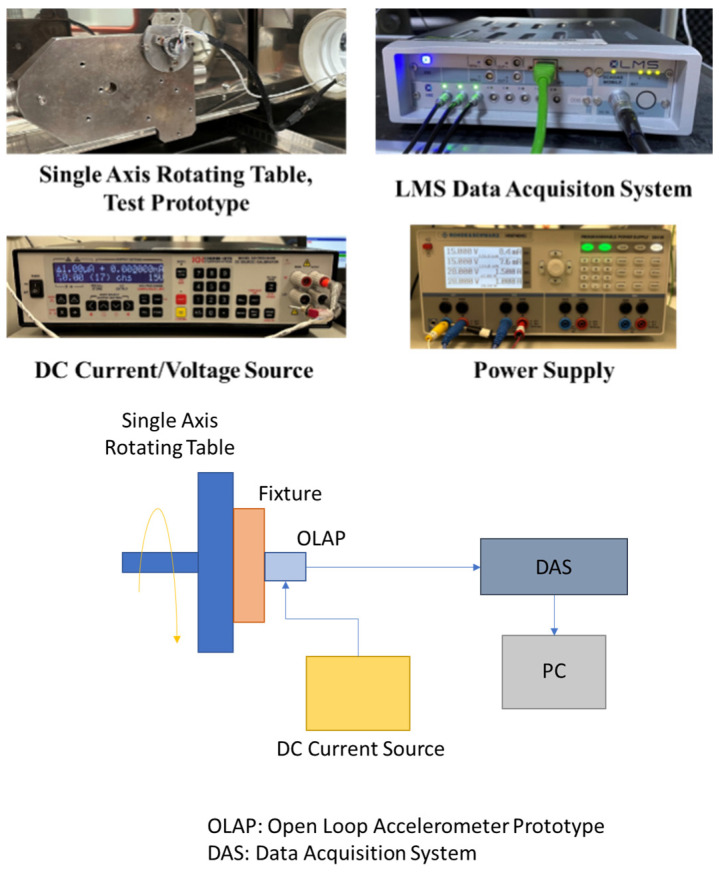
Equipment and the schematics of the open-loop test setup.

The experimental configuration for evaluating the prototype force balance accelerometer in open-loop mode is illustrated in Figure 12. To negate gravitational influence along the sensing axis, the prototype was mounted on a single-axis rotating table for precise orientation adjustment. Coil excitation was provided via a DC-type current/voltage source, while an LMS brand data acquisition system recorded the capacitive pickoff output ($v_{po}$) at a 10 kHz sampling frequency. To ensure thermal stability, the system was energized for one hour before data collection. As the device warms up during this period, the internal temperature distribution gradually stabilizes, leading to a decreasing drift rate and eventual steady state behavior. Repeatability was verified through dual-trial testing, with results exhibiting a maximum variance of 1% across both open-loop and closed-loop configurations. The specific information on instruments used is given below:

- DC Current/Voltage Source: Krohn Hite, Model 523, <8 ppm acceleration output error
- Data Acquisition System: LMS SCM01-V8E, ±10 V input range

**Figure 13:**
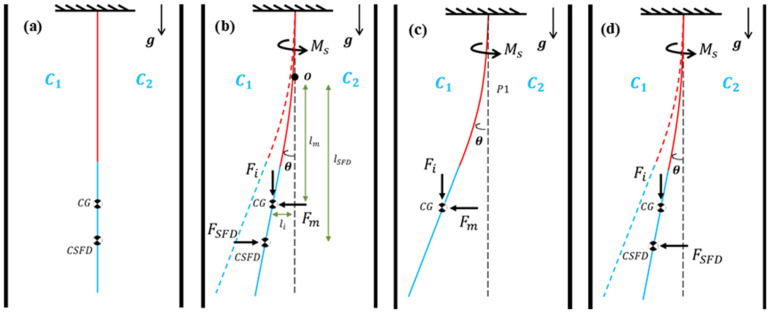
Four phases of the experiments devised for the accelerometer used in open-loop mode: (**a**) phase 1 is the default static position of the pendulum, (**b**) phase 2 consists of the step response to a constant magnetic force excitation applied rapidly, (**c**) phase 3 when magnetic force is kept constant once static equilibrium is reached following phase 2, and (**d**) phase 4 consists of the step response due to rapid removal of magnetic force.

The open-loop performance of the prototype was evaluated by analyzing step responses to varying external coil excitations. Each experimental cycle comprised four distinct phases to characterize the forces acting on the pendulum (Figure 13). These phases, with details given below, systematically evaluate the system’s dynamic equilibrium under controlled electrical loads:

- Phase 1: This is the static equilibrium position, where $C_{1}-C_{2}=0$ with $C_{1}$ and $C_{2}$ as the capacitances of the capacitors of the capacitive pickoff. In this phase, there is no external torquer coil excitation and therefore no magnetic force on the pendulum (Figure 13a). This phase is achieved by manually adjusting the rotating table.
- Phase 2: This is the phase where the transient response is observed and the magnetic force $F_{m}$ is applied as a step input (Figure 13b). Pendulum motion is observed starting from phase 1 until a steady state response is achieved, which then initiates phase 3.
- Phase 3: This phase represents the static equilibrium position where constant external coil excitation and therefore constant magnetic force $F_{m}$ and constant recovery spring torque exist on the pendulum (Figure 13c).
- Phase 4: Starting from phase 3, the second transient step response is observed by removing the magnetic force $F_{m}$ instantaneously (i.e., cutting the coil excitation suddenly) (Figure 13d). Pendulum response is observed starting from phase 3 until the pendulum returns to the static equilibrium configuration, i.e., phase 1.

In the established procedure, phases 1 and 3 define the initial conditions for θ, $F_{m}$ and $F_{i}$, while phases 2 and 4 capture the open-loop transient step responses. These empirical measurements serve to validate the analytical model depicted in Figure 11. Specifically, this data is utilized to verify the amplitude-dependent squeeze film damping (SFD) model previously estimated through finite element analysis (FEA).

Experiments are conducted for 12 different levels of external excitation where step amplitude of the current supplied to torquer coils is different in each case, which are defined as I=1 μA,2 μA,3 μA…12 μA. For I=12 μA, the largest current excitation level, the ratio of maximum amplitude of transverse displacement response of the pendulum (which can be defined as the transverse displacement of the tip of the pendulum relative to the static equilibrium position) to the air gap thickness between pendulum faces and stationary surfaces is calculated to be 83% at the tip of the plate.

**Figure 14:**
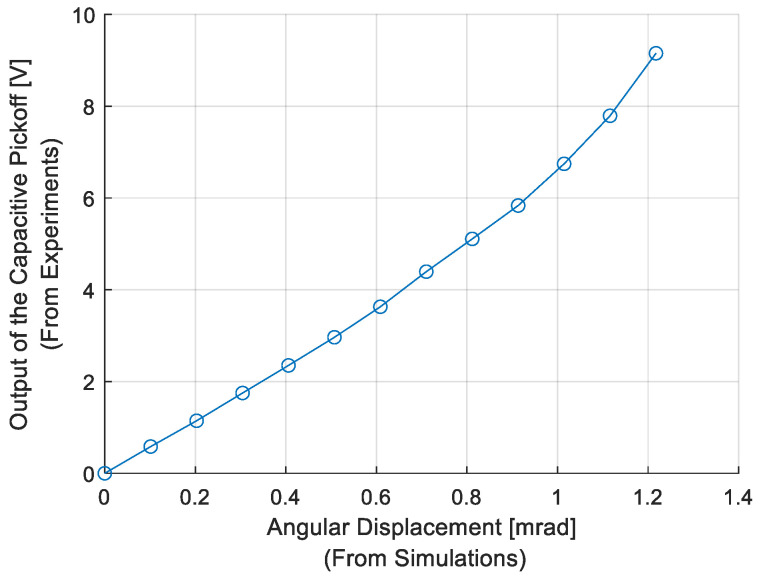
Relation between *θ* and *V_po_*.

In order to verify the model using experimental results, measured quantities are used in two ways. First, by using the steady state θ values from simulations and the steady state $v_{po}(\theta )$ values from open-loop test results, capacitive pickoff gain $K_{po}(\theta )$ is modeled later to be used to convert measured $v_{po}(\theta )$ during step response tests to instantaneous values of θ, the angular displacement response. Here, $v_{po}(\theta )$ values were obtained for different θ values using simulations. By fitting a function to this relationship, $K_{po}(\theta )$ gain was obtained and used as the input to the model. Second, transient responses to the step inputs obtained from experiments and simulations are compared in order to verify the open-loop system model. The settling time of each step response from simulations and experimental results is obtained to make a comparison between them. For phase 2, 98% of the final value and for phase 4, 2% of the initial value are used to determine settling times. The percentage difference between settling times obtained from simulation and experiment is calculated as

(17) $${dif}_{ts}=\frac{{ts}_{sim}^{pos}-{ts}_{exp}^{pos}}{{ts}_{sim}^{pos}}\;*\;100$$

where ${ts}_{sim}^{pos}$ is the settling time of the simulation results and ${ts}_{exp}^{pos}$ is the settling time of experimental results. By using the steady state θ values from simulations and the corresponding steady state $v_{po}(\theta )$ values at the end of phase 2 of open-loop tests (i.e., phase 3), the relation between θ and $v_{po}(\theta )$ is obtained and shown in Figure 14. A polynomial function is fitted to the data in Figure 14, and the capacitive pickoff gain $K_{po}(\theta )$ is obtained experimentally. Then, identified $K_{po}(\theta )$ is used to convert $v_{po}(\theta )$ to θ during step response phases of the experiments.

**Figure 15:**
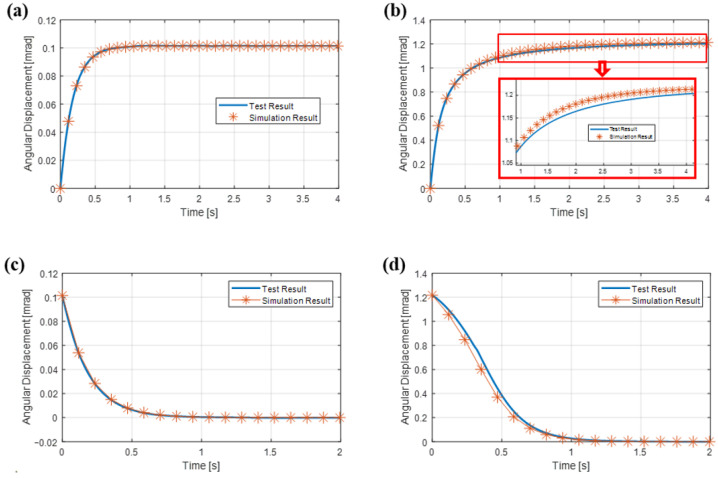
Transient angular displacement responses (step responses to the applied step inputs) from simulations and experiments: (**a**) 1 μA step excitation amplitude used for phase 2, (**b**) 12 μA step excitation used for phase 2, (**c**) 1 μA step excitation amplitude (rapidly reduced to zero to initiate step response) used for phase 4, (**d**) 12 μA step excitation amplitude (rapidly reduced to zero to initiate step response) for phase 4.

Sample transient angular displacement responses (step responses to the applied step inputs) for phase 2 and phase 4 of the experimental scenarios obtained from simulations and experiments are given and compared in Figure 15. In Figure 15a,c, transient angular displacement responses for phase 2 and phase 4 are given, respectively, for the torque coil excitation current of I= 1 μA. In Figure 15b,d, transient angular displacement responses for phase 2 and phase 4 are given, respectively, for the torque coil excitation current of I= 12 μA. The percentage differences between settling times of simulations and experiments for all current excitation levels are given in Table 1. Settling time results are in a high level of agreement for low current excitation amplitudes. As deflection of the pendulum increases, it is observed that the gap between numerical and experimental results widens. For example, ${dif}_{ts}$(%) results are less than 6% up to torque coil excitation current of I= 10 μA, which corresponds to a transverse displacement response of the tip of the pendulum to the air gap thickness ratio of around 50%. Note that for I= 10 μA, the angular displacement amplitude is around 1 mrad, which is close to the maximum amplitude for which the rotational damping coefficient was characterized from previous FEA (Figure 8a). Large differences between experimental and analysis results for I= 11 μA and I= 12 μA may be explained by the fact that when large displacements are reached, stiffness effects caused by the fluid also come into play, changing pendulum dynamics, while our model only accounts for SFD caused by viscous effects. In practical use, the force rebalance loop maintains near-null motion or displaces less than 1 mrad against shock loads, where the model agrees well with experiments and the scale factor is defined via standard low-dynamic calibration. Therefore, these large-displacement deviations do not affect the validity of the usual scale factor calibration and are not representative of intended sensor operation.

## 5. Modeling of the Accelerometer as a Closed-Loop System

In this section, the frequency response and shock response characteristics of the accelerometer as a closed-loop system are investigated. Closed-loop mode is the actual mode of use for force rebalance accelerometers, and the closed-loop system presented in this section represents a full-scale high-fidelity model for the accelerometer being studied. The model will be using the damping model of the SFD identified and verified in previous sections. Using the complete accelerometer model utilizing the verified damping model for SFD, the measurement performance of the accelerometer will be investigated by comparing the frequency response and shock response characteristics of the mathematical and experimental (i.e., physical prototype) models.

In devices using pendulum-type capacitive surfaces, the effective stiffness of the pendulum is generally tried to be kept low in order to reduce nonlinear effects caused by temperature changes and geometric asymmetries [31]. Since the pendulum mass moves freely in open-loop systems, geometric irregularities (as manufacturing of the pendulum components perfectly symmetrically is not possible) at high displacements and more nonlinear effects are observed [31]. Also, for open-loop systems, the position of the pendulum mass cannot be kept constant under high acceleration loads; it may hit opposing surfaces, and the device output becomes saturated. In closed-loop systems, saturation due to mechanical issues can be eliminated [31]. Phase delays observed in the frequency response function of the device at high frequencies can be highly reduced via electronic control in closed-loop systems [31].

**Figure 16:**
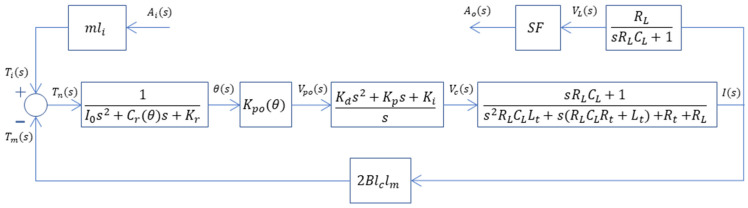
Block diagram of the accelerometer as a closed-loop system.

A block diagram of a single-axis non-gyroscopic closed-loop force rebalance accelerometer is given in [35]. The block diagram used in this study for the system includes the accelerometer and the readout circuit used with it, and is given in Figure 16. The readout circuit is included in the closed-loop model since it has a significant contribution to the dynamic response of the accelerometer. Among the terms given in the block diagram, I(s) is the output current of the controller, $V_{L}\left(s\right)$ is the voltage signal representing the acceleration output of the accelerometer, and $A_{o}(s)$ is the acceleration output of the accelerometer in terms of unit g. For the remaining terms, $R_{t}$ is the total resistance of the coils, $L_{t}$ is the total inductance of coils, $R_{L}$ is the resistance of the load resistor and SF is the scale factor of the system. The scale factor is simply used to convert accelerometer output to the desired unit. MATLAB^®^ 2022b and Simulink^®^ are used to run simulations using the presented model.

The presented model is then used to examine various responses of the system in time and frequency domains. For the frequency domain response analysis, frequency response functions of the system defined between the acceleration input and acceleration output ($A_{i}\left(s\right)-A_{o}(s)$) and harmonic response of the pendulum angular displacement for the same harmonic acceleration input ($A_{i}\left(s\right)-\Theta (s)$) are estimated. Since the model is not a linear time-invariant model due to the displacement-dependent characteristics of terms $C_{r}(\theta )$ and $K_{po}(\theta )$, built-in tools of the software could not be utilized to obtain closed-form frequency responses of the system. Instead, the Simulink^®^ model is run within a loop using a MATLAB^®^ code. In each iteration of the loop, a sinusoidal input signal of constant frequency is fed into the system as a time domain input for a specific duration. The total duration of the signal is determined such that the system responses reach steady state values. Time domain response values (i.e., θ(t) and $a_{o}(t)$) are stored in the MATLAB^®^ environment as outputs of the model. The model outputs stored and the input signals applied are then converted to the frequency domain using the fast Fourier transform (FFT) method. For the acceleration output ($A_{o}(s)$), frequency-dependent complex amplitudes of the output signals are divided by frequency-dependent complex amplitudes of the input signals to obtain frequency response functions in complex form, which are then represented as magnitude and phase values. For the pendulum angular displacement (Θ(s)), the frequency-dependent complex amplitude of this signal obtained from FFT is used to calculate the magnitude of the pendulum angular displacement as a function of frequency for the specific peak amplitude of the acceleration input. The magnitude of the harmonic amplitude of the pendulum angular displacement will be used to justify the importance of the use of displacement-dependent damping behavior of the SFD. The absolute values of the amplitudes of pendulum angular displacement as a function of frequency will directly demonstrate the frequencies at which the amplitude dependence become significant. This process is repeated using different frequencies of input signals to achieve the required frequency resolution across the frequency range of interest, which covers 100 Hz through 2000 Hz (this range covers the low frequencies, where the accelerometer is expected to function and operate accurately, and the expected resonant frequency, which will limit the effective frequency range). For time domain response analysis, the model is used to obtain the responses of the system (i.e., $A_{o}(s)$ and Θ(s) estimated as time-dependent transient signals) to the shock-type acceleration inputs of various amplitudes (i.e., $A_{i}(s)$ defined as a transient shock signal) in the time domain, where model response is obtained via a numerical integration performed by Simulink^®^ in the background. The shock input is defined as a 5 ms-wide rectangular shock waveform with a specified acceleration amplitude. Simulation results are presented in the next section, along with experimental results where model verification experiments conducted for the closed-loop model of the force rebalance accelerometer being studied are also described.

**Figure 17:**
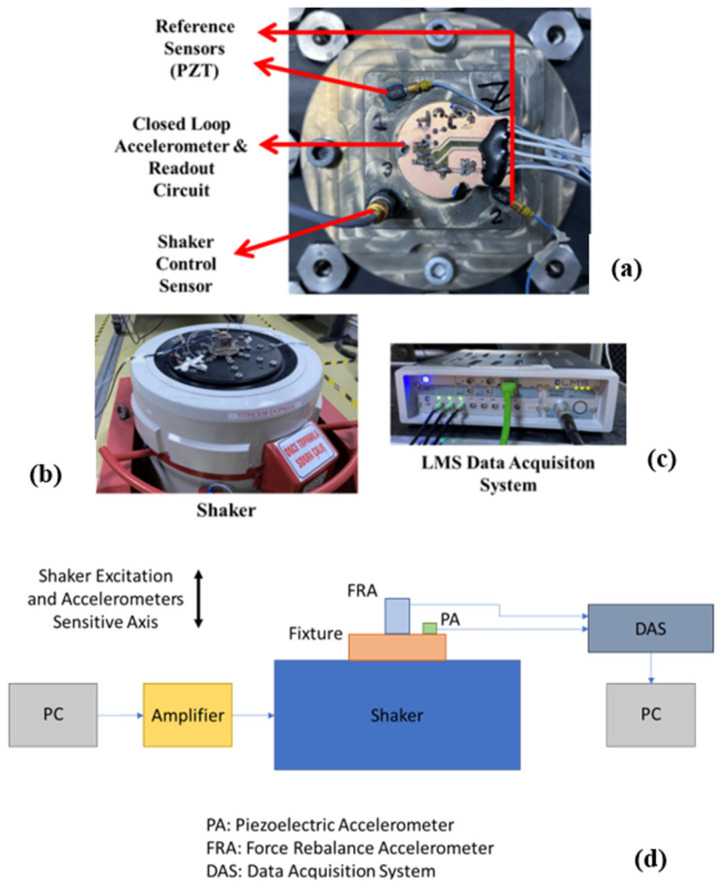
Experimental setup used to test the prototype force balance accelerometer in closed-loop mode: (**a**) prototype force rebalance accelerometer, reference piezoelectric type accelerometer, and piezoelectric accelerometer for shaker controller, (**b**) shaker used to apply the harmonic and shock type acceleration inputs the prototype sensor being tested, (**c**) LMS data acquisition hardware for collecting the test signals, (**d**) schematics of the closed-loop mode setup.

To experimentally verify the closed-loop system model of the force rebalance accelerometer, input–output frequency response functions are obtained from sine sweep tests, and shock responses are obtained from actual mechanical shock experiments. In order to conduct these input-output scenarios, the physical prototype of the force rebalance accelerometer (this time) in closed-loop mode (with all components and the electronics) is utilized, which can be seen in Figure 17a. A vibration shaker (Figure 17b) is used to apply the harmonic and shock-type acceleration inputs to the prototype accelerometer being tested. Piezoelectric (i.e., IEPE)-type accelerometers with large measurement bandwidths (exceeding 10 kHz) and wide measurement ranges are used to generate and measure the reference acceleration values (one for checking the accuracy of the measurement accuracy of the prototype accelerometer, the other to control the motion input generated by the shaker (i.e., armature)). The selection of these sensors ensures non-saturating and reliable measurements, given that the applied acceleration levels are up to approximately 10 g, with up to 20 g expected under resonance conditions, while also providing sufficient bandwidth to cover the frequency range of interest. The prototype force rebalance accelerometer and piezoelectric-type accelerometers are mounted on the same fixture, as close to each other as possible to ensure they measure the same motion. The fixture is designed to be rigid enough not to have a resonance within the target testing frequency range of interest (100–2000 Hz). Additional information on the instruments used in this setup is also listed below:

- Data Acquisition System: LMS, SCM01-V8E (LMS International, Leuven, Belgium) (±10 V Input range).
- Accelerometer: PCB, 352C23 (PCB Piezotronics, Depew, NY, USA) (Range: ±1000 g pk, Frequency range: 2 to 10,000 Hz, Sensitivity (nominal): 5 mV/g, Linearity: ≤ 1%).
- Shaker: Brüel & Kjaer, V850-440 (Brüel & Kjær, London, UK) (Sine Force (pk): 5.7 kN, Max. Acc.: 305 m/s^2^).

Frequency response function measurements in the experiments are conducted for two distinct peak sinusoidal acceleration inputs, with amplitudes of 3 g and 10 g, which represent a moderate-level input and a high-level input, respectively, considering the intended effective measurement range of the prototype accelerometer being investigated. The harmonic acceleration input is applied using a sweep sine-type input signal at a rate of 2 octaves/minute frequency increase within the frequency range of 100 Hz through 2000 Hz, while input acceleration and responses of interest are all recorded. A shaker control system is used to keep the input acceleration peak amplitude constant as the sine testing procedure is implemented. For shock response measurements, two different rectangular shock waveforms, one with a 5 ms width and 3 g amplitude, and the other with a 5 ms width and 10 g amplitude, are applied to the prototype accelerometer to observe initial step responses of the force rebalance accelerometer in the first part of the shock input (i.e., measured response between 0 and 3 ms). Transient data is collected at a sampling rate of 102.4 kHz. By using and processing the input acceleration data measured by the reference piezoelectric accelerometer and the output signals of the force rebalance accelerometer system, frequency responses and shock responses are obtained for the two input amplitude levels. For the same testing scenarios, the simulation results are obtained using the previously described simulation methodology.

**Figure 18:**
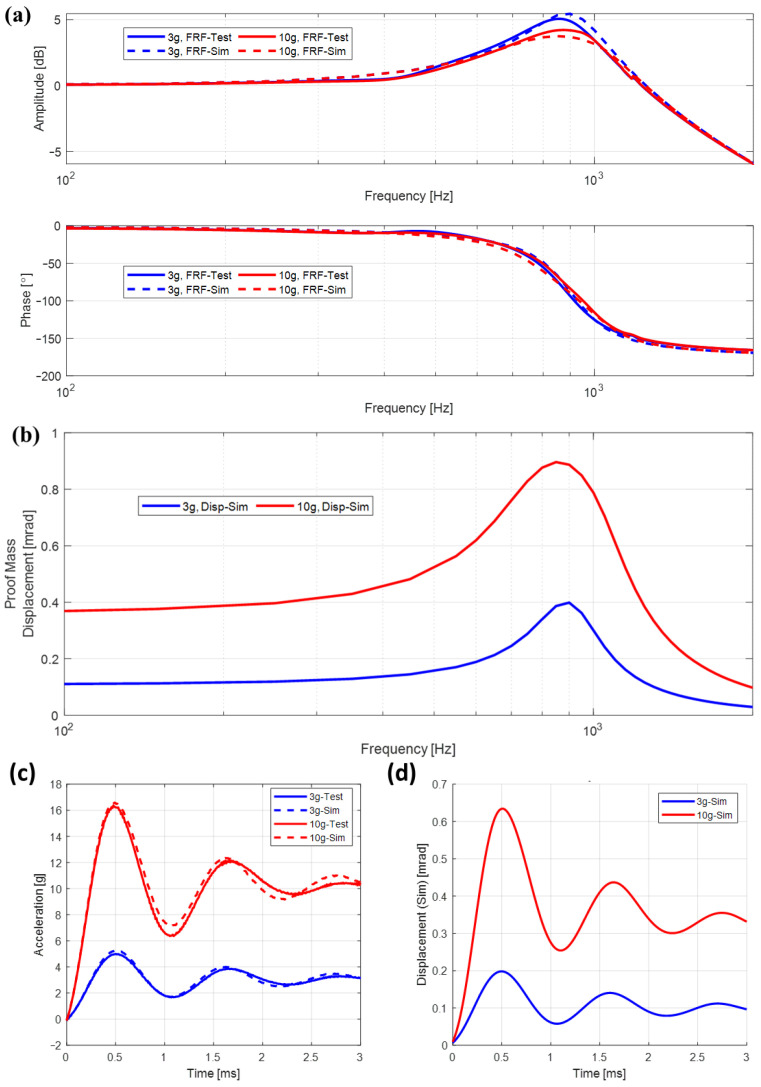
Response plots of the force rebalance accelerometer used in closed-loop mode: (**a**) frequency response function of the acceleration output of the force balance accelerometer to the applied harmonic acceleration input (experimental and simulation results), (**b**) harmonic amplitude of the pendulum angular displacement due to the applied harmonic acceleration input (simulation results only), (**c**) mechanical shock responses of the accelerometer (experimental and simulation results), (**d**) pendulum angular displacement during shock input (simulation results only).

Frequency response functions of the acceleration output signal of the prototype accelerometer for the applied input acceleration of two different levels are given in Figure 18a, along with the simulation results of each test scenario. In Figure 18a, where the acceleration output frequency response function is given in magnitude and phase plots, legends labeled “FRF-Test” and “FRF-Sim” correspond to the magnitudes and phases of the frequency response function of the force rebalance accelerometer in closed-loop mode from the experiment and simulation, respectively. The legend labeled “Disp-Sim” in Figure 18b, where the given plots correspond to the harmonic response amplitude of the pendulum angular displacements caused by the sinusoidal input acceleration, refers to results obtained by simulations. Results for both frequency response function and harmonic response magnitude plots are given for both 3 g and 10 g harmonic input acceleration amplitudes. Frequency response functions of the acceleration output signal of the prototype accelerometer for the applied input acceleration indicate that the prototype accelerometer has a resonance around 900 Hz, which is seen in both simulation and experimental results (Figure 18a). Pendulum angular displacement results (Figure 18b) are only obtained from simulations of the closed-loop system model of the accelerometer since the prototype accelerometer cannot directly output these responses. Pendulum angular displacement results are utilized to investigate the level of angular displacement at different frequencies. Overall, the values of pendulum angular displacement amplitudes increase as the amplitude of the input signal increases, which is somewhat expected. Also, it is seen that the pendulum angular displacement amplitude reaches a peak around the resonance of the accelerometer, while this resonant peak appears moderately damped, which is due to the damping introduced by SFD.

Considering the overall agreement (and more specifically around the resonant peak) of the acceleration output frequency response function magnitude and phase plots from experiments and simulations, it can be said that the displacement-dependent SFD model used in this study represents the actual damping present in the prototype accelerometer accurately. Shock responses of the system output and the pendulum angular displacement to the rectangular wave shocks that are 5 ms wide with 3 g amplitude and 5 ms wide with 10 g amplitude are given in Figure 18c,d, where acceleration output and the pendulum angular displacement plots are presented, respectively, for the first 3 ms. It should be noted that the peak output acceleration exceeds the input excitation levels due to the transient, underdamped dynamic response of the system, leading to overshoot under shock-type inputs. Looking at Figure 18a,c closely, it can be seen that the difference between simulations and experimental results is less than 10% for both frequency response and shock response results, which is an indirect verification of the displacement-dependent damping model for SFD used in this study. Note that the other parameters used in the construction of the model, such as inertia, stiffness, torque coils, and readout circuit system parameters, also play an important role in the agreement observed between simulation and experimental results.

## 6. Conclusions

In conclusion, the SFD in a prototype force rebalance-type accelerometer is modeled mathematically, and this model is successfully verified by comparing the results of full-scale mathematical and experimental models of the accelerometer of interest. The motion of the moving pendulum, which has a partial annular surface, subjected to the SFD originated damping force, is modeled as a single-degree-of-freedom rotational system. When modeling SFD, rarefaction, compressibility, and border effect phenomena are neglected with proper justifications. A displacement-dependent effective rotational damping coefficient representing the SFD is obtained using FEM and FEA. Moreover, the pressure distributions due to SFD are obtained for harmonic motion using FEA, and these results are processed to obtain a displacement-dependent rotational damping coefficient to be used in the dynamic model of the pendulum structure of the accelerometer. The accelerometer is then modeled in full scale for open-loop and closed-loop configurations mathematically and experimentally. Simulation results from the mathematical model and experimental results from the physical prototype of the accelerometer are compared in order to verify the mathematical models built for open-loop and closed-loop modes of use of the accelerometer. Differences between the simulation and experimental acceleration output results of the accelerometer used in open-loop mode to various step acceleration input amplitudes are observed to be less than 6% for up to a transverse displacement response of the tip of the pendulum to the air gap thickness ratio of around 50% and pendulum angular displacement amplitude of around 1 mrad. This indicates that the angular displacement-dependent rotational damping coefficient identified from FEA-based modeling of SFD phenomena could be used up to a transverse displacement response of the tip of the pendulum to the air gap thickness ratio of around 50% and a peak pendulum angular displacement amplitude of about 1 mrad. The difference between results increases dramatically for larger amounts of angular displacements (roughly 1.1 mrad and 12 mrad observed for torque coil excitation currents of I= 11 μA and = 12 μA, respectively), which is an indication of the limitation of the FEA-based model of the SFD. Using the same angular displacement-dependent rotational damping coefficient identified from FEA, along with the model of the other components of the accelerometer, simulation-based acceleration output results for the closed-loop mode are also obtained. The simulation results for the closed-loop system model of the accelerometer are observed to differ from the experimental results up to a maximum value of 10% around the resonant peak for frequency response functions and around the peak response instant for shock response (Figure 18a,c). For the acceleration input amplitudes for these analysis scenarios of the closed mode of the accelerometer used, 3 g and 10 g amplitudes are used to represent moderate and high-level acceleration input situations. The maximum peak pendulum angular displacement amplitudes are slightly below 0.9 mrad for the 10 g input acceleration amplitude. General agreement between the simulation and experimental results demonstrates the strength of the presented SFD and accelerometer modeling methods. This also reflects the fact that the SFD problem considered in this study corresponds to a specific accelerometer geometry and motion characteristic. Therefore, an FEM-based modeling approach is employed to achieve higher accuracy by directly capturing the configuration-dependent and displacement-dependent damping behavior. The strong correlation observed between the simulations and experimental results demonstrates the accuracy of the proposed model. This demonstrated robustness suggests that the modeling and analysis methodology presented in this study can be effectively applied in future design and development efforts for force rebalance-type accelerometers. Its predictive capabilities also offer significant potential for optimizing similar engineering processes and systems. The modeling methodology may not only serve as a valuable tool for current research but also holds promise for facilitating innovation in the intended fields of use. These modeling methods can be utilized for designing a new accelerometer with different performance expectations in terms of sensitivity and frequency range. Possible future work on the presented work may focus on possible improvements in modeling. For example, the accuracy of the model (difference between experiments and simulations) may be further improved by adding higher-order vibration modes to the modeling of the pendulum dynamics, which was modeled as a single-degree-of-freedom rotating system. Compressibility and, thereby, air stiffening effects may also be taken into consideration in SFD modeling in order to increase accuracy, where larger angular displacements are expected to occur.

## Figures and Tables

**Table 1:** Percentage differences in settling times obtained from simulations and experiments for different step current amplitudes applied to torquer coils.

Torquer Current (µA)	1	2	3	4	5	6	7	8	9	10	11	12
${dif}_{ts}^{Case-2}$(%)	−0.2	1.8	3.0	4.0	5.2	5.0	5.9	5.6	4.2	0.6	−8.2	−31.7
${dif}_{ts}^{Case-4}$(%)	0.3	0.8	0.3	0.8	0.5	1.5	1.6	1.9	2.0	2.0	0.8	−3.7

## Data Availability

The data presented in this study are available on request from the corresponding author due to the trade’s confidentiality restrictions.

## Abbreviations

The following abbreviations are used in this manuscript:

CAD	Computer Aided Design
IEPE	Integrated Electronics Piezoelectric
FEA	Finite Element Analysis
FEM	Finite Element Method
FFT	Fast Fourier Transform
FRF	Frequency Response Function
MEMS	Micro Electromechanical Systems
SFD	Squeeze Film Damping
